# Functional status of mechanically ventilated COVID-19 survivors at ICU and hospital discharge

**DOI:** 10.1186/s40560-021-00542-y

**Published:** 2021-03-31

**Authors:** Benjamin Musheyev, Lara Borg, Rebeca Janowicz, Michael Matarlo, Hayle Boyle, Gurinder Singh, Victoria Ende, Ioannis Babatsikos, Wei Hou, Tim Q. Duong

**Affiliations:** 1 Department of Radiology, Montefiore Medical Center and Albert Einstein College of Medicine, 111 E 210th St, Bronx, NY 10467 USA; 2 Renaissance School of Medicine at Stony Brook University, Stony Brook, NY USA; 3 Department of Physical and Occupational Therapy, Renaissance School of Medicine at Stony Brook University, Stony Brook, NY USA; 4 Department of Family, Population and Preventative Medicine, Renaissance School of Medicine at Stony Brook University, Stony Brook, NY USA

**Keywords:** Coronavirus disease 2019, Invasive mechanical ventilation, Functional outcome, COVID-19 sequela, Late effects of COVID-19 infection

## Abstract

**Background:**

A significant number of COVID-19 patients have been treated using invasive mechanical ventilation (IMV). The ability to evaluate functional status of COVID-19 survivors early on at ICU and hospital discharge may enable identification of patients who may need medical and rehabilitation interventions.

**Methods:**

The modified “Mental Status”, ICU Mobility, and Barthel Index scores at ICU and hospital discharge were tabulated for 118 COVID-19 survivors treated with invasive mechanical ventilation (IMV). These functional scores were compared with pre-admission functional status, discharge durable medical equipment, discharge medical follow-up recommendation, duration on IMV, duration post-IMV, demographics, comorbidities, laboratory tests, and vital signs at ICU and hospital discharge.

**Results:**

The majority of COVID-19 IMV patients were not functionally independent at hospital discharge (22% discharged with cane or rolling walker, 49% discharged with durable medical equipment, and 14% admitted to a rehabilitation facility), although 94% of these patients were functionally independent prior to COVID-19 illness. Half of the patients were discharged with supplemental oxygen equipment. The most prevalent medical follow-up recommendations were cardiology, vascular medicine, pulmonology, endocrinology, and neurology with many patients receiving multiple medical follow-up recommendations. Functional status improved from ICU discharge to hospital discharge (*p* < 0.001). Worse functional status at hospital discharge was associated with longer IMV duration, older age, male sex, higher number of comorbidities, and the presence of pre-existing comorbidities including hypertension, diabetes, chronic obstructive pulmonary disease, and immunosuppression (*p* < 0.05, ANOVA).

**Conclusions:**

The majority of IMV COVID-19 survivors were not functionally independent at discharge and required significant follow-up medical care. The COVID-19 circumstance has placed constraints on access to in-hospital rehabilitation. These findings underscore the need for prospective studies to ascertain the short- and long-term sequela in COVID-19 survivors.

## Background

Coronavirus disease 2019 (COVID-19) [1, 2] has infected over 58 million people and killed 1.4 million worldwide (https://coronavirus.jhu.edu, assessed 22 November 2020). The actual numbers are likely to be much higher due to testing shortages and under reporting [3]. There will likely be recurrence and subsequent waves [4]. Moreover, many patients who survived COVID-19 infection will likely have short- and long-term health problems [5]. To date, the majority of published studies related clinical variables to mortality and/or critical illness [6–13]. There is currently no literature that systemically ascertains the functional status of COVID-19 survivors at the time of hospital discharge.

A significant number of COVID-19 patients have been treated using invasive mechanical ventilation (IMV) [1, 2]. Prolonged IMV increases the risk of developing secondary infection, sepsis, and multi-organ failure, which increases patient susceptibility to short- and long-term medical issues [14–16]. The ability to evaluate functional status of COVID-19 survivors early on at ICU and hospital discharge is important because it enables identification of patients who may need medical and rehabilitation interventions. Early intervention has been shown to promote rapid functional recovery and improve quality of life [17, 18].

There are a few clinical tests to evaluate functional status of discharged ICU patients in the hospital settings. Mental status assesses whether a patient is alert and oriented to person, place, time, and situation [19]. The ICU Mobility Scale assesses mobility ranging from being passively rolled in bed, to ambulating independently [20]. The Barthel Index evaluates the level of assistance required to complete basic activities of daily living (ADL) including feeding, toilet transfers and toileting, bathing, dressing, grooming, and stair negotiation [21]. Systematic documentation of functional status in COVID-19 survivors at ICU and hospital discharge could help to anticipate future healthcare needs of COVID-19 sequela [21, 22].

The goal of this study was thus to investigate the functional status of COVID-19 IMV survivors at ICU and hospital discharge. Functional status profiles of IMV COVID-19 survivors included the modified Mental Status Score, ICU Mobility Scale score, and Barthel Index score. In addition, these functional scores were correlated with in-hospital clinical variables, duration on IMV, duration post-IMV, medical follow-up recommendation, discharged durable medical equipment (DME), among others.

## Methods

This retrospective study was approved by the Institutional Review Board with an exemption for informed consent. Our study followed the Strengthening of Reporting of Observational Studies in Epidemiology (STROBE) reporting guidelines for cross-sectional studies (http://www.equator-network.org/reporting-guidelines/strobe/). Data were obtained from 4985 persons under investigation (PUI) presented to the emergency room at Stony Brook University Hospital between 15 March 2020 and 29 June 2020. COVID-19 was confirmed based on a real-time polymerase chain reaction test for severe acute respiratory syndrome coronavirus 2 (SARS-CoV-2) on a nasopharyngeal swab specimen. Patients with incidental COVID-19 findings who were primarily admitted for other major medical indications (i.e., trauma) were excluded. Patients who were still in the hospital at the time of the study and those who were less than 18 years old were also excluded. Inclusion criteria were survivors of COVID-19 who received invasive mechanical ventilation (IMV) and have complete functional status score documentation (*N* = 118).

Demographics, comorbidities, pre-hospital independent status, medical insurance status, laboratories tests, and vital signs were tabulated. Demographic information included age, gender, ethnicity, and race. Chronic comorbidities included smoking, diabetes, hypertension, asthma, chronic obstructive pulmonary disease, coronary artery disease, heart failure, cancer, and chronic kidney disease, among others. Laboratory tests at ICU and hospital discharge included C-reactive protein, D-dimer, ferritin, lactate dehydrogenase, lymphocytes, procalcitonin, alanine aminotransferase, aspartate aminotransferase, and troponin, among others. Vital signs included heart rate, respiratory rate, pulse oxygen saturation [SpO_2_], systolic blood pressure, and temperature at ICU discharge and at hospital discharge.

### Functional scores

The modified Mental Status score (range 0–3) assesses alertness, orientation, and ability to follow command. One point is given if the patient is alert but not oriented, two points are given if the patient is alert and oriented to at least two domains (self, location, time, or situation), and an additional point is given if the patient is able to follow commands. All functional tests were done after patients were extubated or via nonverbal communication. The ICU Mobility Scale (range 0–10) is an 11-item categorical scale that measures the highest level of functional mobility of patients within the ICU setting. The Barthel Index (range 0-100) is an ordinal scale used to measure performance in ADL, consisting of ten variables describing ADL and functional mobility, with a higher number reflecting greater ability to function independently. Due to the isolation precautions placed on patients with COVID-19, therapy sessions were confined to patient rooms. Therapists were not able to accurately assess the "mobility on level surfaces" subscale of the Barthel Index due to these restrictions. Additionally, the ability to negotiate stairs was not consistently assessed across the patient cohort. Stair training was only completed if it was a barrier to discharge home. Therefore, in order to maintain consistency and intergirty to the data set, both the "mobility on level surfaces" and the "stairs" subscales of the Barthel Index were eliminated to more accurately reflect the patient's functional ability, thus changing the range from 0-75. Higher scores indicate higher functioning for all three scores.

Functional scores at ICU and hospital discharge were obtained from patient charts by a team of four occupational therapists and physical therapists. First, this team used a separate set of a dozen ICU patients to reach consensus on how to score patients based on chart review. Chart review included reviews done by occupational therapy notes, physical therapy notes, nursing flowsheets, care management notes, medicine team notes, and speech-language pathology notes if needed. If specific notes and/or information were not available from the actual date of ICU or hospital discharge, the closest note prior to the actual date was used. In situations where patients were re-upgraded to a higher level of care, after being downgraded from the ICU, the ICU discharge date closest to actual hospital discharge was selected. During these chart reviews, COVID-19 diagnosis was confirmed as the final primary diagnosis on the patient chart. Each patient’s medical chart was rated by three independent raters. Inter-rater agreement was evaluated by the interclass correlation analysis.

#### Validation of functional scores

To determine whether the functional status scores derived from chart review reflected scores of the actual tests, a validation study was performed. This was done on a separate group of non-COVID-19 ICU patients (*N* = 18) in which chart review scores and actual test scores were performed independently in a blinded manner from July to September 2020. One rater administered the actual established tests with patients. Three separate and independent raters scored the same patients based on retrospective chart reviews. These three raters did not participate in care of these 18 patients to avoid bias. Inter-rater agreement was also evaluated by interclass correlation analysis.

#### Discharge equipment and notes

The following discharge data were obtained: (i) suggested and actual discharge location (i.e., 1: homecare, 2: rehabilitation facility, 3: long-term care (LTC), or hospice), (ii) discharge equipment (0: none, 1: cane/walker, 2: hospital bed, Hoyer, wheelchair, or commode (durational medical equipment, DME), 3: discharged to rehabilitation facility), (iii) discharge with or without supplemental oxygen equipment, and (iv) discharge follow-up recommendations (i.e., cardiology, vascular medicine, pulmonology, endocrinology, neurology, urology, hematology, surgery, GI, nephrology, psychiatry, ophthalmology, orthopedics/rheumatology, and wound care). Follow-up recommendations of infectious disease and primary care physicians were common to essentially all patients and were not tabulated.

### Statistical analysis

Statistical analysis was performed using SPSS v26 (IBM, Armonk, NY) and SAS v9.4 (SAS Institute, Cary, NC). Suggested discharge locations were compared with actual discharge locations using McNemar’s test. Paired *t* tests were used to compare functional scores, laboratory tests and vital signs at ICU and hospital discharge. Functional scores were compared across different days on IMV, days off IMV, and across different numbers of comorbidities using ANOVA. Multiple regression models were fit to functional scores with demographics and comorbidities as covariates. Backward selection was utilized, and non-significant comorbidities were removed from the final models. For all analyses, a *p* < 0.05 was considered to be statistically significant with correction for multiple comparisons with the false discovery rate where appropriate, unless otherwise specified.

#### Results

At ICU admission, this patient cohort had a median APACHE II and SOFA scores of 16.5 (IQR 12, 21) and 6 (IQR 4, 7), respectively. Demographics, pre-hospital independent status, medical insurance status, laboratory tests, and vital signs at ICU admission and at hospital discharge are summarized in Tables 1 and 2. Aspartate aminotransferase, C-reactive protein, D-dimer, ferritin, lactate dehydrogenase, lymphocyte count, sodium, SpO_2_, procalcitonin, respiration rate, systolic blood pressure, and white-blood cell count were significantly different between groups. Prior to hospital admission, 94% of patients were functionally independent, 4% partially dependent, and 2% dependent. The majority (83%) of patients had medical insurance.

**Table 1:** Demographics

	Frequency	Percent
**Demographics**		
**Gender**		
Male	80	67.80%
Female	38	32.20%
**Ethnicity**		
Hispanic/Latino	36	30.51%
Not Hispanic/Latino	63	53.39%
Unknown/not reported	19	16.10%
**Race**		
Caucasian	52	44.07%
African American	10	8.47%
Asian	8	6.78%
Unknown/not reported	48	40.68%

**Table 2:** Laboratory tests and vitals at ICU discharge and at hospital discharge

	ICU discharge	Hospital discharge	***p*** value
**Laboratory tests**			
Alanine aminotransferase, U/L	91.9 (15.34)	61.6 (6.25)	0.07
Aspartate aminotransferase, U/L	62.3 (12.72)	32.7 (2.33)	0.03^a^
Bicarbonate, mEq/L	23.9 (0.32)	23.15 (0.26)	0.07
C-reactive protein, mg/L	4.62 (0.59)	1.60 (0.20)	1E−06^a^
Creatinine, mg/dL	0.98 (0.10)	0.95 (0.11)	0.80
D-dimer, nmol/L	1307 (128)	940 (90)	0.003^a^
Ferritin, μg/L	1141 (121)	686 (51.5)	1E−05^a^
Hematocrit, %	33 (0.55)	33 (0.49)	0.36
Lactate dehydrogenase, U/L	405 (19)	300 (9.41)	3.8E−06^a^
Lymphocytes, %	12 (0.86)	20 (0.97)	1E−08^a^
Procalcitonin, ng/mL	0.37 (0.06)	0.15 (0.02)	5E−04^a^
Sodium, mEq/L	143 (0.45)	140 (0.37)	3E−08^a^
White blood cells, G/L	12 (0.38)	9.5 (0.34)	3.6E−07^a^
**Vitals**			
SpO_2_ , %^b^	97 (0.16)	96 (0.16)	3.2E−08^a^
Heart rate, bpm	90 (1.45)	88 (1.16)	0.15
Respiration rate, rate/min	22 (0.41)	19 (0.28)	1.7E−10^a^
Systolic blood pressure, mmHg	135 (1.70)	122 (1.42)	3.3E−10^a^
Diastolic blood pressure, mmHg	72 (0.82)	72 (0.78)	0.57
Temperature, ^o^C	37 (0.37)	37 (0.02)	0.21
**Functional scores**			
Mental Status Score	2.33 (0.09)	2.85 (0.05)	1.4E−09^a^
ICU Mobility Scale	1.20 (0.17)	5.78 (0.26)	7.8E−34^a^
Barthel Index Score	10.25 (1.29)	38.21 (2.06)	9.37E–24^a^

^a^Statistical significance. Values in parentheses are SEM

^b^SpO2 is not reliable because of the missing FiO2 data which were not reliably recorded at ICU and hospital discharge

**Fig. 1:**
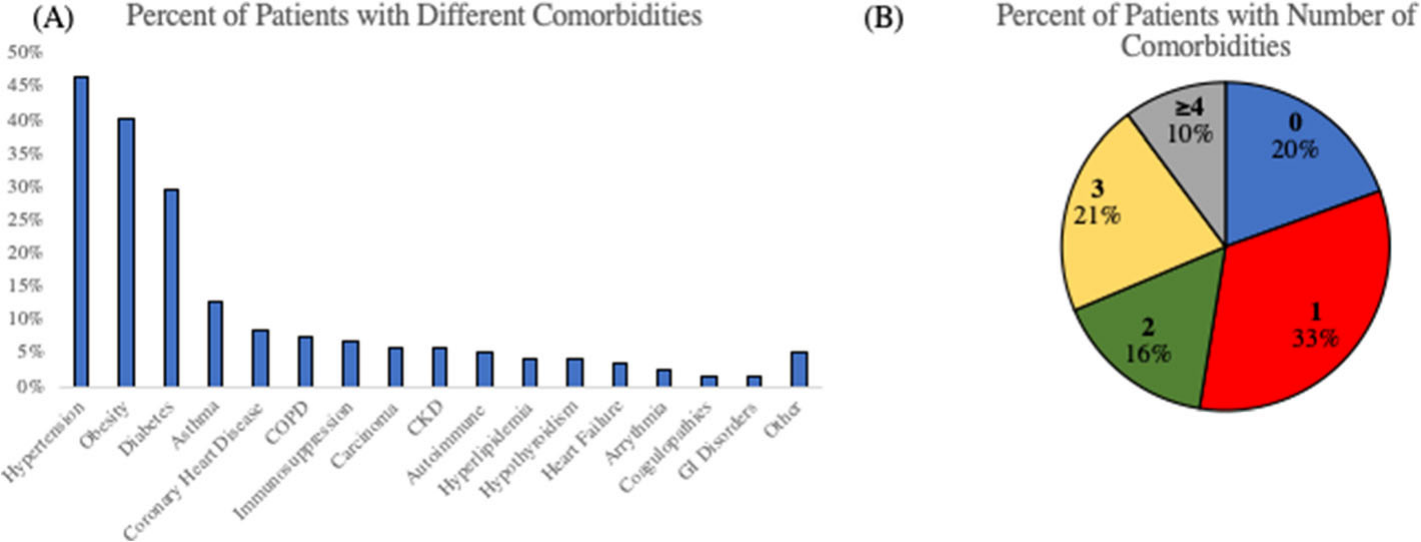
**a** Prevalence of comorbidities of IMV COVID-19 survivors. **b** Percent of patients with different comorbidities and number of comorbidities

The most prevalent comorbidities were hypertension (47%), obesity (40%), diabetes (30%), and asthma (13%) (Fig. 1a). In our group, 19% of patients had none, 33% had one, 16% had two, 21% had three, and 10% had four or more comorbidities (Fig. 1b).

**Fig. 2:**
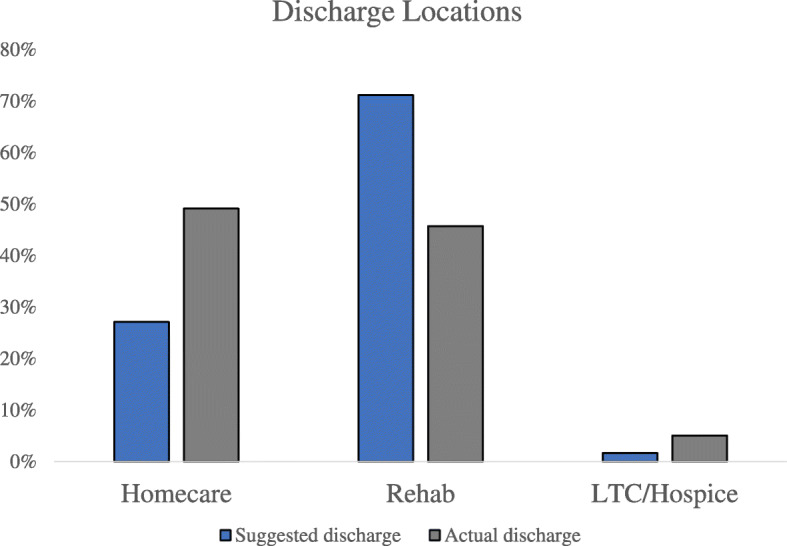
Recommended and actual discharges of IMV COVID-19 survivors. *p < 0.001 (McNemar’s test)

The percentages of patients with recommended discharge to homecare, rehabilitation, LTC/hospice were 27%, 71%, and 2%, respectively (Fig. 2). The percentage of patients with actual discharge to homecare, rehabilitation, LTC/hospice were 49%, 46%, and 5%, respectively. Of those who received rehabilitation recommendation, 17% of insured patients and 5% of uninsured patients elected homecare instead. Significantly more discharged patients elected homecare over acute and sub-acute rehabilitation facilities against recommendations (*p* < 0.001, McNemar’s test).

**Fig. 3:**
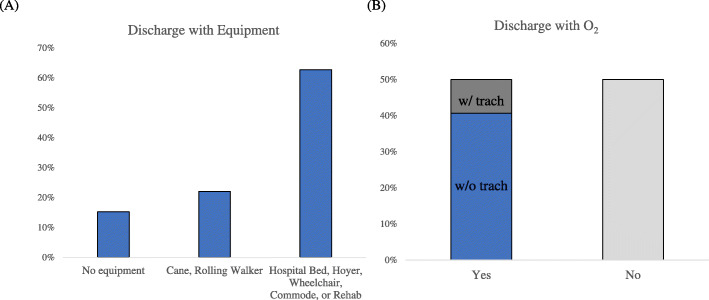
**a** Percentage of patients discharged: (i) with no equipment, (ii) with cane or rolling walker, (iii) with hospital bed, Hoyer, wheelchair, commode (also referred to as durable medical equipment, DME), and/or discharged to a rehabilitation facility. **b** Patients discharged (a) with or (b) without supplemental oxygen equipment

The percentage of patients who were discharged (i) with no equipment, (ii) with cane or rolling walker, (iii) DME or rehabilitation facility were, respectively, 15%, 22%, and 63%, (Fig. 3a). Half (50%) of the patients were discharged with supplemental oxygen equipment of which 9% had a tracheotomy and 41% did not (Fig. 3b). The remaining 50% of patient were discharged without oxygen equipment.

**Fig. 4:**
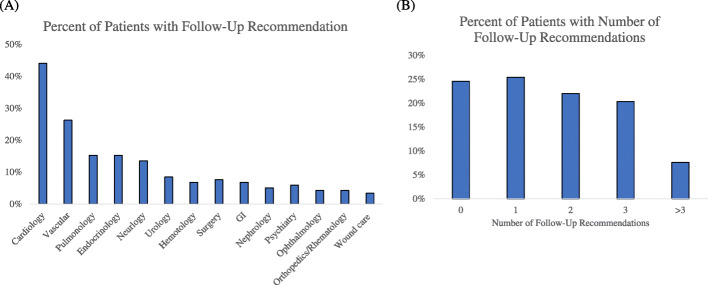
**a** Histogram of medical follow-up recommendations, and **b** percentages of patients with number of follow-up recommendations

The major medical follow-up recommendations included cardiology (44%), vascular medicine (26%), pulmonology (15%), endocrinology (15%), neurology (14%), urology (8%), hematology (7%), surgery (8%), and gastroenterology (GI) (7%) (Fig. 4a). In our group, 25% of patients had none, 25% had one, 22% had two, 20% had three, and 8% had at least four medical follow-up recommendations (Fig. 4b).

### Functional scores

For the validation study (*N* = 18, non-COVID19 ICU patients), the intra-class correlation coefficients between “actual test” scores and “chart review” scores for the modified Mental Status, ICU Mobility Scale, and Barthel Index scores were, respectively, 1.000, 0.997, and 0.987, among the three independent raters. These high inter-rater agreements suggested that functional scores were well documented on patients’ chart.

**Fig. 5:**
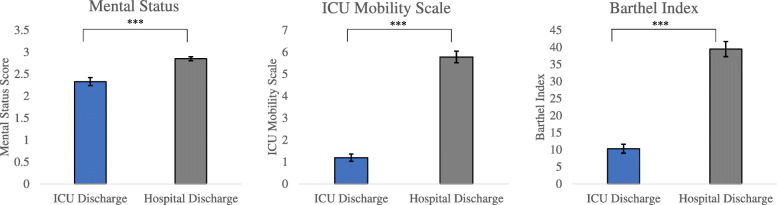
**a** Mental status, **b** ICU mobility, **c** Barthel scores at ICU (blue) and hospital (gray) discharge. Error bars are SEM. *** indicates *p* < 0.0001

About half (53%) of the IMV total patients (*N* = 118) received only physical or occupational therapy, and 47% received both. The inter-rater agreement of three raters by interclass correlation coefficients were 0.948, 0.954, and 0.976 for modified Mental Status, ICU Mobility Scale, and Barthel Index scores, respectively. Figure 5 shows the functional status scores at ICU and hospital discharge. All patients showed significant improvement in functional scores at hospital discharge relative to the scores at ICU discharge (*p* < 0.0001 all three scores, paired *t* tests). Functional status of IMV patients was abnormal at hospital discharge.

**Fig. 6:**
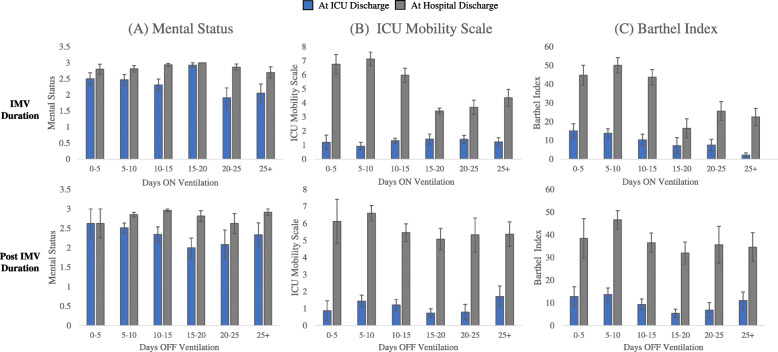
Duration (**a**) on IMV and (**b**) post-IMV for mental status, ICU mobility, and Barthel scores at ICU and hospital discharge. Duration on IMV correlated with ICU Mobility Scale score (*p* < 0.001, ANOVA) and Barthel Index discharge (*p* < 0.001, ANOVA) at hospital. There were no other significant correlations

Figure 6 shows the functional status scores at ICU and hospital discharge versus binned duration on and off IMV. A shorter duration on IMV was correlated with a better ICU Mobility Scale and Barthel Index scores at hospital discharge (*p* < 0.001 for both scores, ANOVA) but not at ICU discharge (*p* > 0.05, ANOVA). Duration on IMV were not correlated with Mental Status scores at both ICU and hospital discharge (*p* > 0.05, ANOVA). There were no correlations with off IMV (*p* > 0.05, ANOVA).

**Fig. 7:**
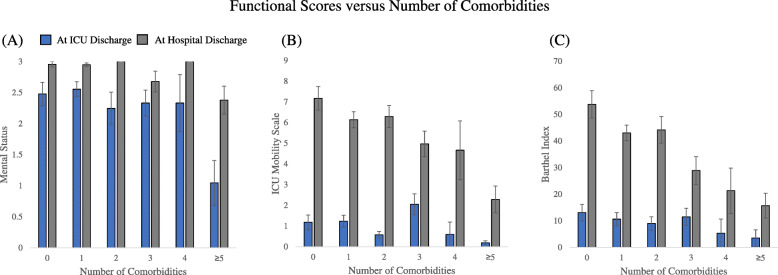
**a** Modified Mental Status (range 0–3), **b** ICU Mobility Scale (range 0–10), **c** Barthel Index (range 0–75) scores versus number of comorbidities. Functional scores correlated with the number of comorbidities at hospital discharge (*p* < 0.05, ANOVA) but not at ICU discharge (*p* > 0.05)

Functional scores decreased with increasing number of comorbidities at hospital discharge (*p* < 0.05, ANOVA) but not at ICU discharge (*p* > 0.05, ANOVA) (Fig. 7). Table 3 shows the correlation of ICU Mobility Scale and Barthel Index scores at hospital discharge with demographics and comorbidities. Some functional scores were significantly correlated with the age, sex, and the presence of pre-existing comorbidities including hypertension, diabetes, chronic obstructive pulmonary disease, and immunosuppression (*p* < 0.05, ANOVA). Correlations for comorbidities that had < 6% prevalence were not analyzed as they were unreliable.

**Table 3:** Regression coefficients (standard errors) of functional status scores at hospital discharge with demographics and comorbidities

Hospital discharge			
	Mental Status	Mobility Score	Barthel Index
Demographics			
Age		− 0.06 (0.02) **	− 0.88 (0.15) ***
Gender	0.26 (0.08) ***	1.44 (0.50) **	
Comorbidities			
Obesity			
Hypertension		− 0.98 (0.48) *	
Diabetes			− 10.13 (3.99) *
Asthma			
Coronary heart disease			
COPD	− 0.45 (0.14) **		
Immunosuppression	− 0.29 (0.14) *		
Carcinoma	− 0.47(.15) **		
Chronic kidney disease			

*COPD* chronic obstructive pulmonary disease

* indicate *p*<0.05, ** indicates *p*<0.01, *** indicates *p*<0.001

## Discussion

This study investigated the functional status of COVID-19 IMV survivors at ICU and hospital discharge. The major findings were (i) the majority of patients were functionally independent prior to COVID-19 illness, but not at hospital discharge, (ii) half of patients were discharged with supplemental oxygen equipment, (iii) the most prevalent medical follow-up recommendations were cardiology, vascular medicine, pulmonology, endocrinology, and neurology with many patients receiving multiple medical follow-up recommendations, and (iv) worse functional status at hospital discharge was associated with longer IMV duration, older age, male sex, higher number of comorbidities, hypertension, diabetes, chronic obstructive pulmonary disease, and immunosuppression. To our knowledge, this is the first study that systemically evaluates functional status of COVID-19 survivors and correlate with clinical variables at ICU and hospital discharges.

COVID-19 re-infection concerns at rehab facilities or a desire to go home weighed in on patient’s and/or caretaker’s decisions [23]. Per Executive Order 202.30 issued on 10 May 2020 by NYS Gov. Andrew Cuomo, nursing rehabilitation facilities required patients to be tested COVID-19 negative to be admitted. This might have contributed to a longer length of stay in the hospital as an appropriate and safe discharge plan was not feasible until a negative test was obtained. It is also possible that insurance status might have influenced such decision, as patients without insurance require approval to be considered as a charity case. Our data showed that insurance status did not play a significant role in electing homecare versus rehabilitation facility in this patient cohort. These factors along with the COVID-19 circumstance placed significant constraints on patients receiving rehabilitation and in a timely manner.

Our findings suggest that most patients were not functionally independent, and many still had significant unresolved medical issues at hospital discharge. Follow-up studies are important to ascertain long-term outcomes and anticipate healthcare needs for COVID-19 survivors. Based on the referral prevalence, cardiac and vascular embolism issues were more concerning overall than pulmonary issues [24, 25]. Studies have shown that SARS-CoV-2 virus enters host cells via the angiotensin-converting enzyme 2 (ACE2) receptors which are found to have a relatively high density in the heart [26]. Similarly, hospitalized COVID-19 patients are at higher risk of future blood clots such as deep vein thrombosis and pulmonary embolism [27], and thus there is a need for follow up with vascular medicine to monitor such potential events.

In our cohort, about half of the IMV patients received only physical or occupational therapy. Under normal circumstances, most IMV patients would have received in-hospital physical or occupational therapy prior to discharge. During COVID-19, physical and occupational therapy sessions were limited due to environmental barriers as patients were to remain in their rooms, medical equipment as patients were tethered to oxygen lines and telemetry monitors, and decreased staffing availability. Concerns about cross infection and unclear guidelines early in the COVID-19 pandemic might have contributed to lower rates of in-hospital rehabilitation. Although functional scores of IMV survivors showed improvement relative to those at ICU discharge, many of these patients were clearly physically and functionally impaired at hospital discharge.

Longer IMV duration was associated with worse ICU Mobility Scale and Barthel Index scores at hospital discharge. This is not unexpected because IMV patients with longer IMV duration were sedated, received neuromuscular blocking agents for longer period of time, consistent with known effects of IMV [28]. It is surprising that IMV duration was not correlated with Mental Status Score. A possible explanation is that the Mental Status Score has a narrow dynamic range (0–3). The majority of the patients had a mental score close to 3 on average, indicating that most patients were relatively alert mentally by this measure at the time of hospital discharge. IMV duration was correlated with ICU mobility score and Barthel score at hospital discharge. It is not surprising that IMV duration was correlated with Barthel score and ICU mobility score because it is generally expected that longer duration of mechanical ventilation and worse functional outcomes.

Older age and male sex were significantly correlated with a worse functional score. Older age and male sex have previously been associated with more severe illness and higher mortality rate [6–10]. Some functional scores at discharge were significantly correlated with hypertension, diabetes, chronic obstructive pulmonary disease, and immunosuppression. These findings are not surprising because hypertension and diabetes have previously been associated with critical illness and mortality [6–10]. Our findings that mental status was negatively correlated with the presence of COPD, immunosuppression, and carcinoma are consistent with expectation because patients with these conditions were often sicker and thus more likely to have a worse mental status. However, this finding needs to be interpreted with caution because Mental Status Score has a small dynamic range (0–3). Prospective studies are needed to confirm this finding.

Prolonged ICU stay in general has been associated with higher risk for ICU-acquired weakness, delirium and other medical issues including post-intensive care syndrome (PICS) and Chronic Fatigue syndrome (CFS) [22, 29–31]. These patients experienced general ICU-acquired weakness which affects functional scores. ICU patients receive sedation medication and neuromuscular blocking agents. Many patients also received dexamethasone, a corticosteroid. Common risk factors for PICS, categorized by limitations in physical and cognitive functioning after a prolonged critical illness, include acute respiratory distress syndrome (ARDS), prolonged mechanical ventilation, delirium, and multi-organ system failure [31]. These risk factors are associated with COVID-19 infection, and thus healthcare providers must consider PICS when treating patients with COVID-19 as research has shown that symptoms may persist for years after acute illness [29–31]. Patients with COVID-19 face long ICU stay which puts them at risk of prolonged bed rest, leading to muscle weakness and deconditioning, as well as decreased pulmonary function which impacts overall endurance and activity tolerance [29–31]. When combined with the social isolation needed to mitigate risk of infection to others, many patients could face psychological distress and post-traumatic distress disorders [22]. Many survivors of PICS and ARDS are at risk of more cognitive impairments, specifically with sustained attention, memory, and executive functioning [29]. Future prospective studies should delve deeper into these domains using standardized cognitive assessments such as the Confusion Assessment Method (CAM-ICU) which monitors ICU-acquired delirium. Early interventions in ICU patients during hospitalization and post-hospital discharge has been shown to be effective to promote physical, cognitive and psychological health, speed up functional recovery [17, 18, 32] and improve quality of life [22]. Unfortunately, the need to mitigate risk of cross infection under the COVID-19 circumstance has placed significant constraints on rehabilitation interventions and in timely manner. Our findings suggest that the COVID-19 survivors are at a high risk of developing long-term medical sequela which could increase healthcare utilization downstream. Many of these patients might not be able to fully return to work, resulting in additional societal burdens.

### Limitations and future perspectives

This study has several limitations. This is a study from a single hospital. Multi-site studies are needed to achieve generalizability of these findings. As with any retrospective study, there are potential unintentional data selection bias and confounding variables that were not taken into account. The data extracted from the electronic medical record was also limited by the retrospective nature of this study. This study only investigated COVID-19 survivors treated with IMV. Future studies will include general floor COVID-19 patients. Reduced functioning in our cohort could be short- or long-term and thus follow-up studies of COVID-19 IMV survivors are important. Future prospective studies could include additional functional measures.

### Conclusions

This study investigated the functional status of IMV COVID-19 survivors at hospital discharge. The majority of IMV COVID-19 survivors were not functionally independent at discharge and might require significant follow-up medical care. The COVID-19 circumstance requiring precautions to mitigate cross infection risk, places significant constraints on patients receiving rehabilitation in a timely manner. Our findings underscore the need to perform prospective studies to ascertain short- and long-term sequela in COVID-19 survivors. It would not be surprising that COVID-19 sequela will increase healthcare expenditure and reduce work productivity for years to come.

## Data Availability

The datasets used and/or analyzed during the current study are available from the corresponding author on reasonable request.

## Abbreviations

ACE2: Angiotensin-converting enzyme 2
ADL: Activities of daily living
APACHE II: Acute physiology and chronic health evaluation II
ARDS: Acute respiratory distress syndrome
CAM-ICU: Confusion assessment method
CFS: Chronic fatigue syndrome
COPD: Chronic obstructive pulmonary disease
COVID-19: Coronavirus disease 2019
DME: Discharged durable medical equipment
GI: Gastroenterology
ICU: Intensive care unit
IMV: Invasive mechanical ventilation
LTC: Long-term care
PICS: Post-intensive care syndrome
Rehab: Rehabilitation facility
SARS-CoV-2: Severe acute respiratory syndrome coronavirus 2
SOFA: Sequential organ failure assessment
SpO_2_: Pulse oxygen saturation
Trach: Tracheotomy
